# Dynamic modulations of effective brain connectivity associated with postural instability during multi-joint compound movement on compliant surface

**DOI:** 10.1007/s00221-025-07039-2

**Published:** 2025-03-03

**Authors:** Tim Lehmann, Anton Visser, Tim Havers, Daniel Büchel, Jochen Baumeister

**Affiliations:** 1 Exercise Science & Neuroscience Unit, Department of Exercise & Health, Faculty of Science, Paderborn University, Paderborn, Germany; 2 Department Fitness and Health, IST University of Applied Sciences, Duesseldorf, Germany

**Keywords:** Postural control, Multi-joint compound movement, Compliant surface, Effective brain connectivity

## Abstract

Random fluctuations in somatosensory signals affect the ability of effectively coordinating multimodal information pertaining to the postural state during movement. Therefore, this study aimed to investigate the impact of a compliant surface on cortico-cortical causal information flow during multi-joint compound movements. Fifteen healthy adults (7 female / 8 male, 25.9 ± 4.0 years) performed 5 × 20 repetitions of bodyweight squats on firm and compliant surface. Motor behavior was quantified by center of pressure (CoP) displacements, hip movement and the root mean square of the rectus femoris activity. Using source space analysis, renormalized partial directed coherence (rPDC) computed subject-level multivariate effective brain connectivity of sensorimotor nodes. Bootstrap statistics revealed significantly decreased medio-lateral CoP displacement (*p* < 0.001), significantly increased velocity of medio-lateral hip motion (*p* < 0.001) as well as significantly lower rectus femoris activity (*p* < 0.01) in the compliant surface condition. On the cortical level, rPDC showed significantly modulated information flow in theta and beta frequencies for fronto-parietal edges (*p* < 0.01) only during the concentric phase of the movement. The compliant surface led to increased difficulties controlling hip but not center of pressure motion in the medio-lateral plane. Moreover, a decreased activation of the prime movers accompanied by modulations of effective brain connectivity among fronto-central nodes may point to altered demands on sensorimotor information processing in presence of sensory noise when performing bodyweight squats on compliant surface. Further studies are needed to evaluate a potential benefit for athletic and clinical populations.

## Introduction

In the sports context, closed-kinetic chain exercise on compliant surface - is a key element of prevention and rehabilitation training programs for challenging postural control, the neural ability to effectively monitor body position and alignment in space (Ageberg and Roos 2015; Behm et al. 2015). In that respect, previous research has already shown that exercise combined with surface instability may support neural information processing, as expressed by improved multi-joint coordination, muscular co-activations and anticipatory postural adjustments (Behm et al. 2015; Saeterbakken et al. 2019). While in quiet stance, postural sway is primarily controlled through small reflexive adjustments of the ankle and hip muscles (Winter 1995), multi-joint compound movements extend these principles by incorporating more complex postural challenges and neuromuscular coordination across several joints for simultaneously stabilizing the body in multiple directions (Caterisano et al. 2002; Robertson et al. 2008).

In theory, the sensorimotor network controls postural equilibrium during multi-joint compound movements through complex spinal and supraspinal neural circuits. It continuously integrates multimodal information from afferent sensors and subsequently generates efferent motor commands for compensating inherently unstable dynamics of the body (Kuo 2005; Shumway-Cook and Woollacott 2012, pp. 162–173). Recent evidence has suggested that the cerebral cortex may utilize ascending sensory information for adapting subcortical postural action systems responsible for the coordination of synergistic muscle responses (Peterson et al. 2016). While visual and vestibular sensory information is used to monitor the interrelationship between body segments relative to the environment, somatosensory information predominantly serves to control postural stability, describing the relationship between the center of mass and the base of support (Horak 2006; Shumway-Cook and Woollacott 2012, pp. 162–173). However, random and unpredictable fluctuations or disturbances in sensory signals, referred to as sensory noise, may affect the ability of effectively coordinating multimodal sensory information about the postural state (Faisal et al. 2008). In presence of a compliant base of support, somatosensory noise accumulates owing to randomness of mechanical surface fluctuations, as proprioceptive information from lower extremity muscle spindles or tactile receptors does not appear coherent with instantaneous changes in body orientation relative to the environment (Faisal et al. 2008; Kiers et al. 2012; van Dieën et al. 2015). As a result, increased sway and acceleration of the body, as well as altered muscle synergies become apparent (Gebel et al. 2019; Büchel et al. 2021). The external disturbances of neural processing may therefore require temporal reweighting of sensory information to adjusts the relative importance of different sensory information. Eventually modifications of the supraspinal directed neural information flow within a distinct network of sensorimotor areas might be required to efficiently cope with dynamic postural demands (van der Kooij and Peterka 2011; Pasma et al. 2012; Wittenberg et al. 2017).

In that respect, mobile neuroimaging studies utilizing electroencephalography (EEG) have already demonstrated frequency-specific topological modulations of oscillatory cortical dynamics in relation to instability-evoked postural responses (Mierau et al. 2017; Solis-Escalante et al. 2019; Varghese et al. 2019; Gebel et al. 2020; Lehmann et al. 2020; Büchel et al. 2021; Sherman et al. 2021). Increased theta band (4–7 Hz) oscillations were found to reflect a general brain integrative mechanism allowing to modulate sensory information or adapting to unexpected perturbations (Edwards et al. 2018; Gebel et al. 2020). Alpha band oscillations (8–12 Hz) were associated with a global alertness and with task-specific sensorimotor processing during postural tasks (Del Percio et al. 2009; Cheron et al. 2016; Gebel et al. 2020; Hülsdünker et al. 2020; Büchel et al. 2021), whereas beta band activity (13–30 Hz) was linked with modulations of motor commands in response to dynamic or unpredictable postural challenges (Jacobs and Horak 2007; Wittenberg et al. 2017). Although several cortical areas appear to be involved in postural control processes, a subset of fronto-central cortical regions has frequently been attributed a particular functional relevance for handling challenges to postural equilibrium (Wittenberg et al. 2017). It has been suggested that the prefrontal cortex, anterior cingulate cortex, supplementary motor area, premotor cortex and posterior parietal cortex may interconnect through neural oscillations in theta (4–7 *Hz*), alpha (8–13 *Hz*) and beta (13–30 *Hz*) frequencies in order to process sensorimotor information relevant for postural control (Mierau et al. 2017; Solis-Escalante et al. 2019, 2020; Varghese et al. 2019). In temporal relation to preparatory or compensatory postural responses, adjacent functional connections (edges) within this postural control network show increased connectivity strength (Mierau et al. 2017; Varghese et al. 2019). The causal information flow (effective connectivity, Friston 2011) between areas (nodes) associated with postural control may therefore sign the sensorimotor system to handle externally evoked postural instability. However, while previous findings were predominantly derived from rather static balancing tasks (Wittenberg et al. 2017), the investigation of rather dynamic compound exercises on compliant surface may provide further insight into cortico-cortical dynamics associated with postural instability.

Since initial findings from Kenville and colleagues (2020) have already demonstrated that mobile EEG is applicable for identifying cortical dynamics in different phases of a bodyweight squat, the aim of the present study was to explore the effects of postural instability on cortico-cortical causal information flow during multi-joint compound movements on mechanically unstable surface. Based on current observations (Mierau et al. 2017; Solis-Escalante et al. 2019; Varghese et al. 2019), it was hypothesized that postural stability may decrease and effective connectivity within edges of adjacent fronto-central nodes may increase due to higher postural demands while moving on compliant surface. In order to properly link cortical phenomena to concomitant postural stability and muscle activity, a combined mobile brain and body imaging approach was deemed expedient for opening new perspectives on the complex postural control dynamics in presence of somatosensory noise.

## Materials and methods

### Subjects

Initially, eighteen healthy and physically active young adults (9 female / 9 male, 25.8 ± 4.3 years, 171.7 ± 8.6 *cm*, 67.3 ± 10.7 *kg*) were recruited to participate in the present study (Table 1) and gave written consent to their participation. Participants completed a health history questionnaire to determine potential restrictions or limitations regarding their participation. In case of any medical diagnosis which may influence postural stability, including traumatic cartilage injuries, degenerative changes of the knee joint, chronic ankle instability or previous surgery to the knee/ankle joint, participants were excluded from the study. With regards to the EEG measurements, medication intake of neuroactive or psychoactive drugs, implanted cardiac pacemaker, metal implants in the head or face, skull abnormalities or fractures, history of a neurological/psychological diseases, recurring or severe headaches/migraine, concussion within the past 6 months, previous heart or brain surgery, seizures at any time or history of epilepsy, served as further exclusion criteria. Collectively, all subjects actively participated in different sports (running, cycling, fitness, athletics, and badminton), but reported no previous experiences with exercising on compliant surface. The study protocol was approved by the local research and ethics committee.

**Table 1:** Descriptive statistics of the study sample

	Initially recruited	Included in final analysis
*Demographics*		
Sex (female / male)	9 / 9	7 / 8
Age (years)	25.8 ± 4.0	25.9 ± 4.1
Height (cm)	171.7 ± 8.4	171.1 ± 9.0
Weight (kg)	67.3 ± 10.4	66.6 ± 11.2
*Anamnesis*		
Dominant leg (left / right)	7 / 11	6 / 9
Experience resistance exercise (y / n)	7 / 11	6 / 9
Experience balance exercise (y / n)	3 / 15	3 / 12
Experience unstable surface exercise (y / n)	0 / 18	0 / 15

### Experiment

**Fig. 1:**
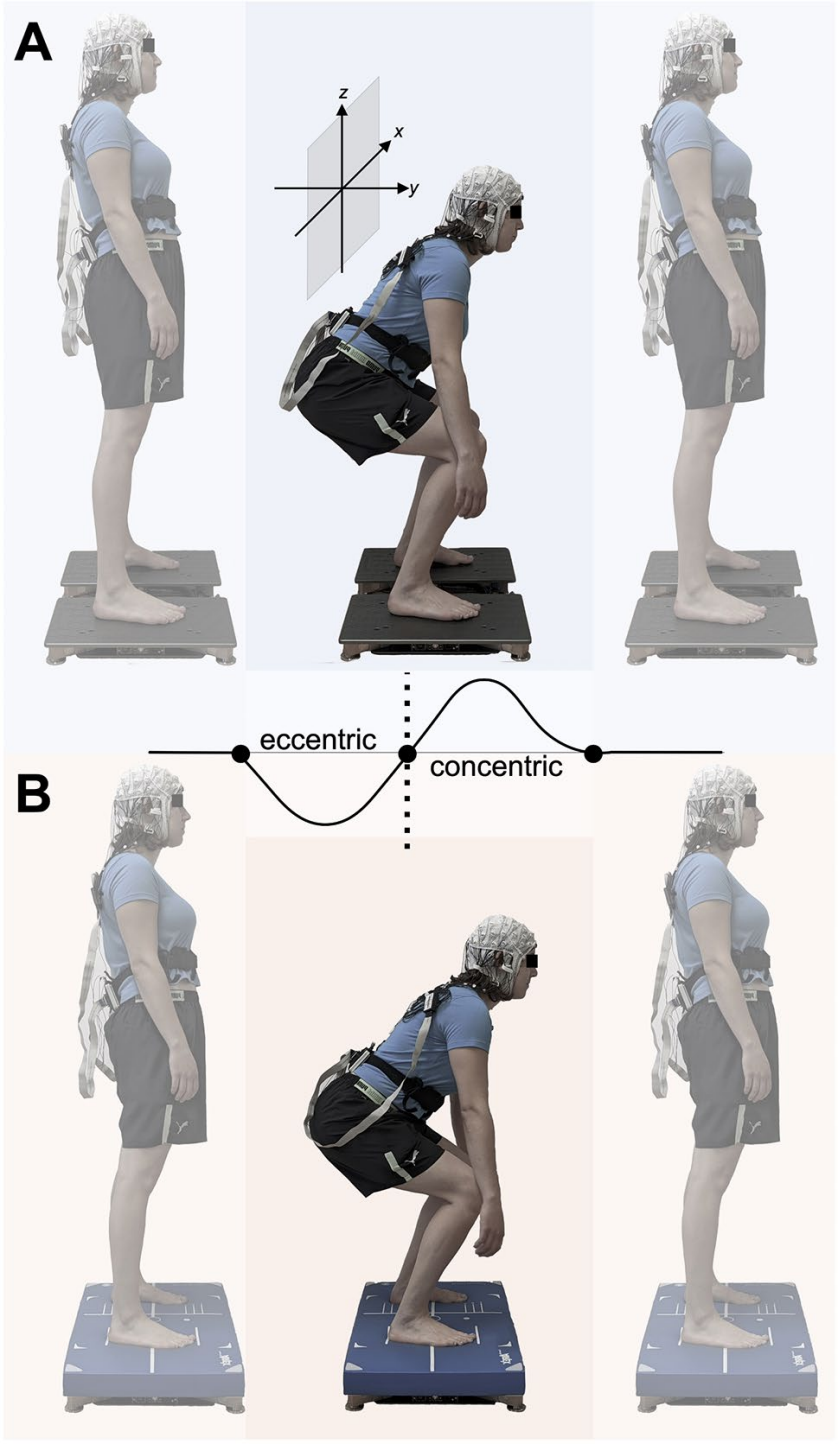
Experimental task, starting in standing position on either firm (**A**) or compliant (**B**) surface, followed by the downward movement (eccentric) and a consequent elevation (concentric) of the hip. Relative directions of hip motion are shown as *x* (medio-lateral), *y* (antero-posterior) and *z* (vertical)

A single-group crossover design was used to investigate the effects of surface instability on postural sway, hip motion, muscle activity and cortico-cortical connectivity during bipedal multi-joint compound movements. For this purpose, synchronized accelerometry, posturography, electromyography (EMG) and mobile EEG were recorded in a single experimental session. All data recordings were controlled and synchronized in time using a custom-built interface, developed in a cross-platform engine (Unity3D, Unity Technologies, USA). Prior to the measurement, subjects were informed about the experimental procedures and equipped with mobile brain and body imaging sensors. The experiment commenced with a standing resting-state in which participants remained in stable, fully extended bipedal stance for 90 *s* in front of a white wall to familiarize with the equipment. Afterwards, participants completed ten blocks of 20 consecutive bodyweight squats on either firm or compliant surface in randomized block order (total of 100 trials per condition). The compliant surface was thereby created by a commercially available 6.0 cm thick foam pad (Balance Pad Elite, Airex AG, Switzerland), as it has been shown to produce reliable instability in postural control tasks (Lin et al. 2015). At the beginning of the test, subjects were instructed to adopt a comfortable stance width in which a knee flexion angle of approximately 90 degrees could be achieved at the end of the downward movement (knee/hip flexion, eccentric phase). This stance width was kept equal between surface conditions and blocks. Further instructions included maintaining a slight lumbar lordosis and to avoid raising the heels from the supporting surface at the transition point to the upward movement (knee/hip extension, concentric phase). Movement execution and gaze fixation was standardized across subjects by using a visual cue displayed on a ground-level screen placed 50 *cm* apart from the force platforms. The movement cadence of one trial was 3 *s* baseline– 1.5 *s* eccentric– 1.5 *s* concentric– 1 *s* post movement period (Fig. 1). Furthermore, to avoid conflicting sway or muscle artifacts in the EEG, subjects were instructed to keep their arms relaxed alongside the body and to maintain a fixed declined tilted head position (Kenville et al. 2020a). For limiting confounding influences of cumulative peripheral fatigue, breaks between blocks were kept at least at 60 *s* and adapted to the individual needs of the subjects. As physical fatigue could potentially affect postural sway and cortical activity (Gebel et al. 2022), individual strenuousness of the overall experimental protocol was determined at the end of each experiment. For this purpose, the session rate of perceived exertion scale (Foster et al. 2001) was used to evaluate the overall subjective effort based on a scale from 1 to 10 (*no exertion* to *maximal exertion*).

### Accelerometry

In order to validate the exact movement cadence, a purpose-built belt was used to place a triaxial accelerometer sensor integrated in the EEG amplifier (LiveAmp64, Brain Products, Germany) on the sacrum of participants. Accelerometry data was recorded in three directions (*x*, *y*, *z*) at a sampling rate of 500 *Hz*. To determine the onset movement periods (eccentric / concentric) and the movement termination points for all following analyses, a custom-built detection algorithm (MATLAB, v.R2020, Mathworks Inc., USA) was used. The algorithm incorporated data smoothing of the sum vector in *x* and *z* directions using Savitzky-Golay filtering based on the quadratic polynomial fitted over moving windows of 750 samples. This smoothing procedure was selected for the detection, as it effectively reduces noise in data with rapid signal variations, while simultaneously preserving maxima, minima and the widths of these peaks (Rigoberto et al. 2012).

Local maxima in the input vector, distinguished by their intrinsic height and relative peak-to-peak separation, were identified. Abrupt linear changes in the mean and slope of the surrounding signal could be assigned to the onset of eccentric and concentric phases, as well as movement termination. The resulting timestamps were used to assess deviations (mean latency in *s*) between the actual movement execution and the prescribed movement cadence displayed on the screen. Additionally, the timestamps were used for epoching of the mobile brain and body imaging data.

For the analysis of hip motion in medio-lateral (*x*), antero-posterior (*y*) and vertical (*z*) directions, the raw data was downsampled to 100 *Hz*. To minimize a gravity component linked with slow body sway and to reduce signal noise, a fourth-order zero-phase Butterworth filter with a high-pass cutoff frequency of 0.01 *Hz* and a low-pass cutoff frequency of 2 *Hz* was applied (Rigoberto et al. 2012; Alkathiry et al. 2018). The mean velocity (*mm/s*) of hip motion was then calculated from the filtered acceleration signal by means of the trapezoidal numerical integration using the MATLAB *cumtrapz* function. The magnitude of the normalized path length *(NPL*, *mG/s*, where 1 *mG* is 1/1000 of the gravitational acceleration constant) was calculated as the sum of the absolute path length distances normalized to their sequential time length (Alqahtani et al. 2017). With respect to the posturographic metrics, the NPL was only calculated for medio-lateral (*x*) and antero-posterior (*y*) directions.

### Posturography

The assessment of postural stability was based on center of pressure (CoP) trajectories acquired from a dual force plate system (ForceDecks FDLite, VALD Performance, Australia) at a sampling rate of 1000 *Hz*. Data processing was performed using the vendor-supplied ForceDecks software (ForceDecks, VALD Performance, Australia) and involved an adjustment of the vertical ground reaction forces for the additional load of the foam pad. Furthermore, the data was low-pass filtered with a fourth-order Butterworth filter at a cutoff frequency of 10 *Hz* and finally downsampled to 100 *Hz* sampling rate. Subsequent analysis included the parameter *mean velocity* (*mm/s*) as the total excursion in relation to the length of the trial. Further, *antero-posterior (AP) and medio-lateral (ML) range* were determined as the maximum CoP displacement (*mm*) in either direction (Duarte et al. 2011).

### Electromyography

Neuromuscular activity of the prime mover during squats– the m. rectus femoris muscles (Clark et al. 2012)– was recorded bilaterally using a portable electromyography (EMG) system (Ultium, Noraxon Inc., USA) with dual-octagon-shaped electrodes (size of 5 × 2.5 *cm* and 2 *cm* interelectrode distance). Skin preparation was done by shaving, abrading and cleaning to ensure optimal data quality. Dual electrodes were mounted on the muscle belly of the rectus femoris according to the SENIAM recommendations (Hermens et al. 2000) splitting the distance between the superior iliac part of the anterior spine and the superior part of the patella. Adhesive tape and straps (myoMotion TM Straps, Noraxon USA Inc., USA) were used to prevent electrodes from detaching during movement execution. The raw EMG data was recorded at a sampling rate of 2000 *Hz* and wirelessly transmitted to MR3 software (myoMuscleTM, Noraxon USA Inc., USA). The input impedance of the amplifier was > 100 *MΩ*, analog bandpass filtering was applied in the frequency range of 10–500 *Hz* and common-mode rejection was set at > 100 *dB*.

For increasing the signal to noise ratio, data was preprocessed in MATLAB, including a low-pass Chebyshev Type I filter with a pass-band frequency at 200 *Hz* and stop-band at 220 *Hz*. According to recommendations for dynamic and non-vigorous movements (De Luca et al. 2010), an additional fourth-order Butterworth high-pass filter with a cut-off frequency of 20 *Hz* was used. In order to reduce computational costs of the analysis, data was downsampled to 500 *Hz* (Ives and Wigglesworth 2003). After full-wave rectification, the pre-processed EMG data were converted to root mean square (RMS) using a moving average of 0.2 *s*. Finally, differences of muscle activation (amplitude in *mV*) between firm and compliant conditions were evaluated using the mean RMS envelope of both legs during the eccentric and concentric phase of the movement (van den Tillaar et al. 2019).

### Electroencephalography

Cortical activity was continuously recorded from 65 active Ag/AgCl electrodes (actiCap, Brain Products, Munich, Germany) placed according to the extended international 10–10 system. The data was transmitted through a wireless transmission path (LiveAmp64, Brain Products, Munich, Germany), digitally amplified at a sampling rate of 500 *Hz* and online low-pass filtered at 200 *Hz*. The amplifier was placed in a strap system at the level of the sacrum to allow unrestricted mobility. Electrode impedance was kept below 25 *kΩ* to ensure an appropriate signal-to-noise ratio. All electrodes were online referenced to an FCz reference montage including AFz as the ground electrode. To capture the exact position of each electrode site for each individual subject, a 3D electrode localization scanning system (Captrak, Brain Products GmbH, Germany) was utilized. For this purpose, reference markers on the preauricular points as well as the nasion served as anatomical landmarks relative to the EEG electrodes.

All EEG data processing was done with the EEGLAB open source toolbox (Delorme and Makeig 2004) and complemented by customized MATLAB scripts. Initially, the individually digitized electrode information was fitted to the datasets, including circumference and shape of the head, as well as the Cartesian and polar coordinates for each electrode position (*x*, *y*, *z*, *θ*, *φ*, *r*). Afterwards, the FCz reference channel was restored. Timepoints of interest detected from the accelerometer timeseries were then imported to the EEG event structure. Sinusoidal line noise (50/100 *Hz*) was removed (Mullen 2012) and a basic finite impulse response filter with a bandpass of 3–30 *Hz* was applied. Finally, the data was re-referenced to common average and downsampled to 256 *Hz*. In order to avoid confounding influences on connectivity measures, eBridge (Alschuler et al. 2014) was used for identifying channels that are linked by low-impedance electrical bridges. Additionally, *clean_rawdata* (Kothe and Jung 2015; Chang et al. 2020) was used to remove channels with transient or high amplitude noise (line noise criterion: 4), poor correlation with adjacent channels (channel criterion: 0.8), prolonged flatline channels (flatline criterion: 5) and to apply automatic subspace reconstruction for removing and interpolating non-stationary high-amplitude bursts with large variance (burst criterion: 10). In the case of channel rejections, the data were re-referenced to common average again. For the quantification of cortical network dynamics, the continuous EEG data was divided into epochs ranging from − 3000 *ms* to 4000 *ms* relative to movement onset, in order to achieve a solid frequency resolution and narrow frequency bins for a precise identification of individual frequency components, as well as a sufficient amount of datapoints for the subsequent source localization (Onton et al. 2006).

**Fig. 2:**
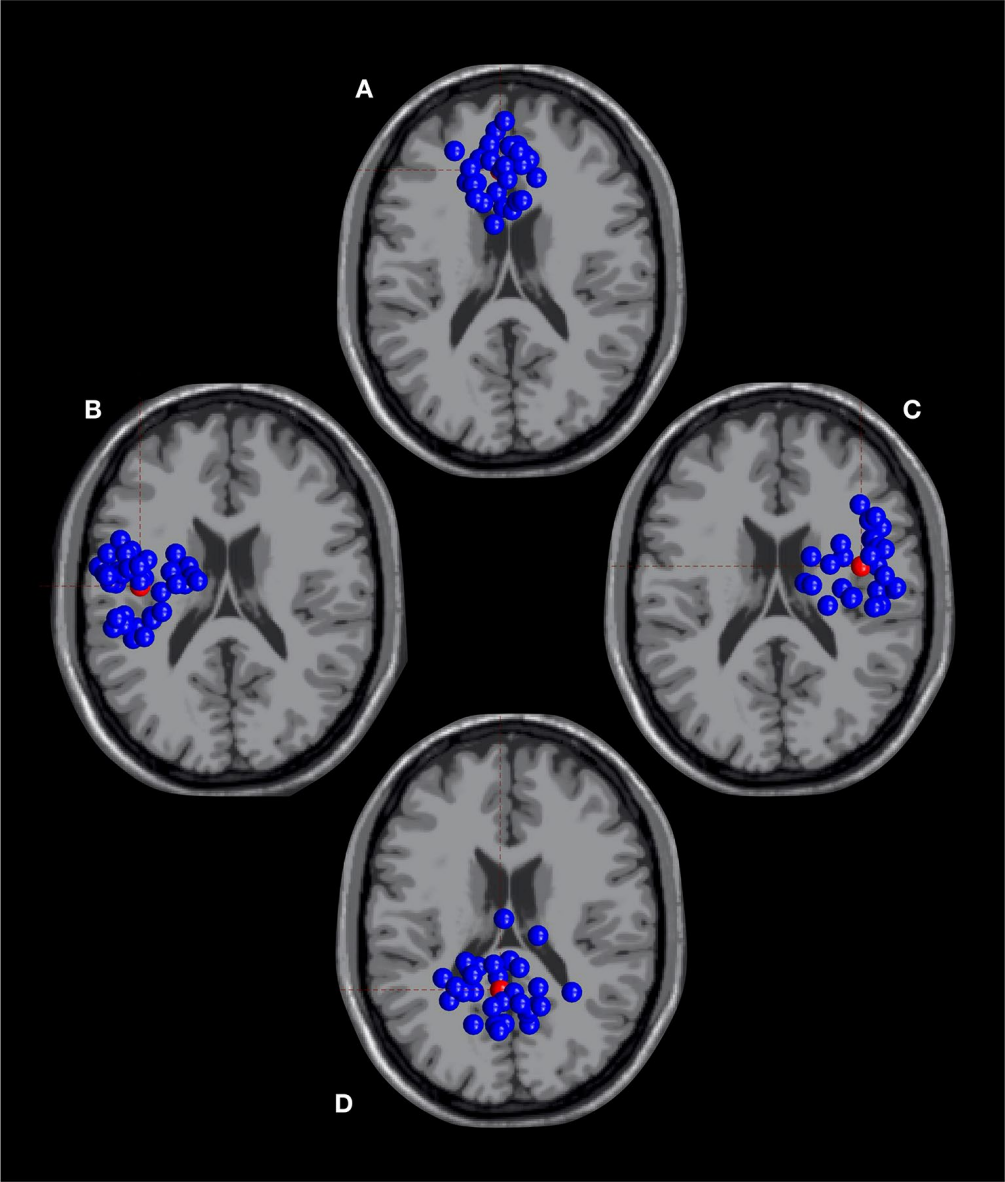
Original clusters of independent components as a base for selecting subjects for further analysis: fronto-central (**A**), motor left (**B**), motor right (**C**) and posterior parietal (**D**)

To decompose the data into maximally independent components (ICs) of electrophysiological activity, adaptive mixture independent component analysis (AMICA, Palmer et al. 2011) was applied to the pre-processed scalp recording. A default MNI head model of the DIPFIT function (Oostenveld and Oostendorp 2002) was then used to estimate equivalent dipole locations of the decomposed sources. The set of ICs was then classified based on their individual scalp topography, equivalent dipole location and activity power spectrum (Pion-Tonachini et al. 2019). Subsequently performed k-means clustering (Fig. 2) revealed 4 sensorimotor clusters of interest (Ø 17 subjects / 30 ICs) based on common spatial features. The clusters were selected with respect to dipole locations in proximity to the fronto-central, motor and posterior parietal cortex, as these cortical regions have previously been associated with postural control and surface instability (Wittenberg et al. 2017; Gebel et al. 2020; Kenville et al. 2020a; Büchel et al. 2021). Only subjects contributing at least one IC to all four clusters were eligible for further analysis. The resulting sample for the connectivity analyses therefore consisted of 15 participants, all meeting the criteria of comparable dipole locations and high-quality data.

In order to generate an effective connectivity model with equal number of input variables, multiple ICs per subject within one cluster (Ø 1.81 ± 0.91 ICs / subject / cluster) were pooled by normalizing and averaging their scalp maps, recomputing weights and sphering matrix, as well as recomputing ICA activations.

For the analysis of event-related causal information flow between the multivariate IC time series, renormalized partial directed coherence (rPDC) was calculated across sources. A parametric multivariate autoregressive model (MVAR) was computed across the ICA-derived effective sources of sensorimotor activity and subsequently fitted by Viera-Morf algorithm utilizing the EEGLAB groupSIFT plugin (Loo et al. 2019; Koshiyama et al. 2020; Jurgiel et al. 2021). To fit the MVAR model for each dataset with respective ICs for each condition (firm / compliant), a grand-average optimum model order of 10, a sliding Hamming window with a length of 1.0 *s* and a step size of 0.02 *s* was selected, finally generating 30 log-scaled frequency bins from 4 to 30 *Hz*. The model for both conditions was then validated based on statistical whiteness (0.80 ± 0.05 / 0.79 ± 0.05), precent data consistency (85.42 ± 2.42 / 85.56 ± 2.36), the parameter-to-datapoint ratio (0.02 ± > 0.0) and model stability index (-0.07 ± 0.01).

Previously estimated dipole locations were transformed into probabilistic dipole densities by using a 3D Gaussian kernel with full-width at half maximum (FWHM) of 20 mm truncated at 3 sigma, in order to resolve the spatial variability between subjects. Thereby, the conversion to group anatomical regions of interest (ROI) was based on the automated anatomical label atlas (AAL, Tzourio-Mazoyer et al. 2002) and required at least 80% of the subjects contributing non-zero dipole densities. Here, 9 of the original 76 AAL graph nodes demonstrated overlap in both conditions (firm / compliant), which ultimately created a connectivity matrix depicting causal flow differences generated from the ROI-to-ROI pairwise dipole densities weighted by rPDC.

### Statistical analysis

All statistical analyses were performed in MATLAB, incorporating comparisons of firm vs. compliant surface conditions for accelerometry, posturography, EMG and EEG data. Due to the properties of the neurophysiological data from EMG and EEG, movement periods (eccentric / concentric) were also analyzed separately. Bootstrap statistics were used to assess the effects of surface instability on the dependent variables (except for EEG). By drawing 2000 bootstrap samples with replacement from the mean differences between variables, the probability of observing a significant difference at *p* ≤ 0.05 was estimated. Additionally, effect sizes were calculated using Cohen’s *d* (*d*) to evaluate the magnitude of differences between conditions, interpreting *d* ≥ 0.2 as small, *d* ≥ 0.5 as medium, and *d* ≥ 0.8 as large effects (Cohen, 1988).

Computation of source-level connectivity statistics was performed using the previously mentioned groupSIFT framework. For each graph edge, pixelwise two-sample *t*-tests between the two conditions were performed on each rPDC time-frequency map masked at uncorrected *p* < 0.01. Groups of neighboring pixels with surviving *t*-statistics were combined, representing significant differences between conditions in the time-frequency domain. The resulting *t*-score maps were corrected using permutation tests (2000 iterations, *p* < 0.01) and the generalized family-wise error rate (FWER) control as a cluster-level correction (Nichols and Hayasaka 2003; Groppe et al. 2011).

## Results

### Sample

The final dataset was composed of 15 subjects (7 female / 8 male, 25.9 ± 4.0 years, 171.1 ± 9.0 *cm*, 66.6 ± 11.7 *kg*). Out of the 15 subjects, only 3 reported prior experiences with balance training, whereas none of the subjects has previously exercised on compliant surfaces. Furthermore, the average rate of perceived exertion after completing the entire protocol was 4.0 ± 1.8, corresponding to *comfortable / moderate* exertion (Borg 1998).

### Accelerometry

The latencies of movement execution relative to the visual cues did not show significant differences between conditions for the onset of the eccentric period (-0.015 ± 0.046 *s*, *p* = 0.19, *d* = 0.33) and the point of movement termination (0.006 ± 0.085 *s*, *p* = 0.77, *d* = 0.07), but was significantly higher for the onset of the concentric movement period on compliant compared to firm surface (0.037 ± 0.069 *s*, *p* = 0.03, *d* = 0.54).

**Fig. 3:**
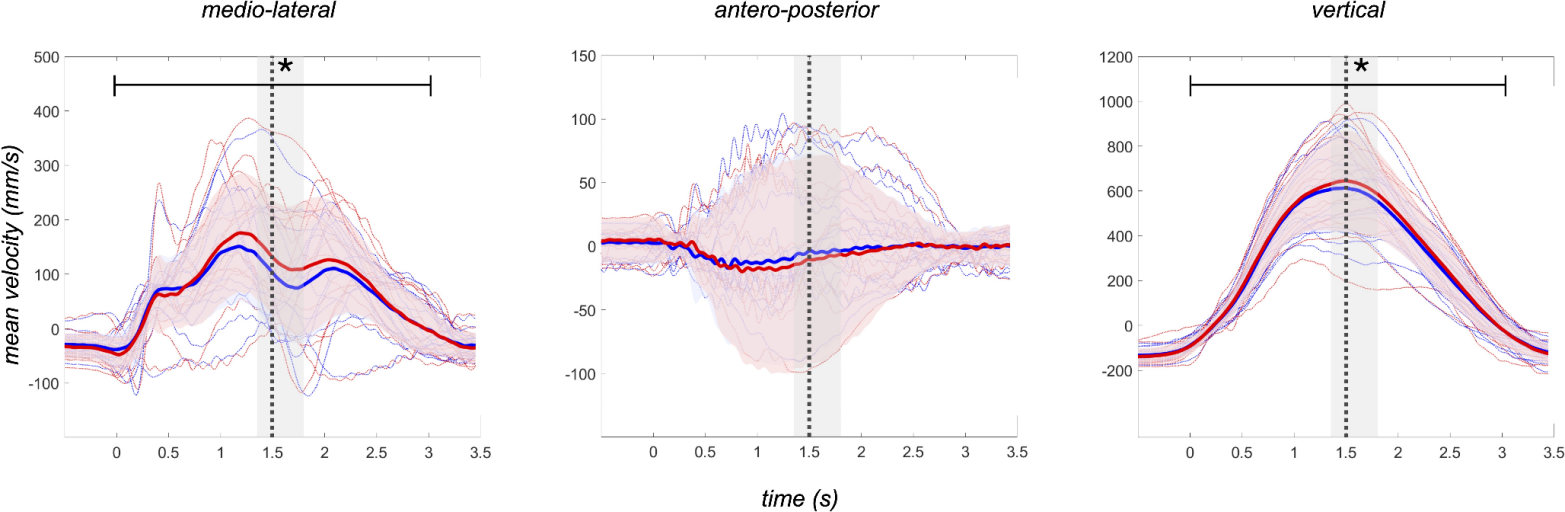
Mean velocity (*mm/s*) of hip movement under firm (*blue line*) and compliant (*red line*) conditions in medio-lateral, antero-posterior and vertical direction. Dotted lines separate eccentric and concentric phases, with timing variations shown as shade. The distribution of individual samples is displayed as dashed lines, together with the standard deviation (*shade*) throughout the entire trial length. Significant differences at *p* < 0.05 between conditions are marked with asterisk (*)

The analysis of hip movement (Fig. 3) revealed significantly higher mean velocity in medio-lateral (15.99 ± 18.09 *mm/s*, *p* = 0.001, *d* = 0.88) and vertical direction (20.53 ± 28.32 *mm/s*, *p* = 0.004, *d* = 0.72) on compliant surface, whereas mean velocity in antero-posterior direction was not significantly different between conditions (2.83 ± 5.77 *mm/s*, *p* = 0.05, *d* = 0.49).

**Fig. 4:**
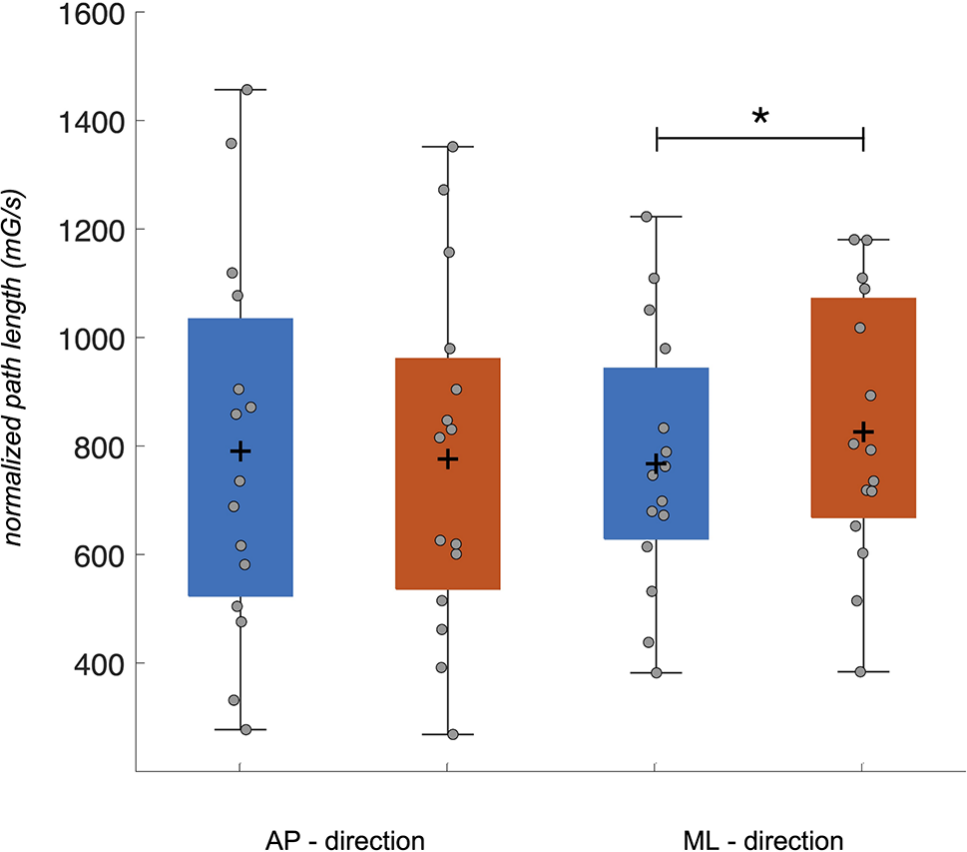
Magnitude of hip movement, depicted as the normalized path length (*mG/s*) under firm (*blue*) and compliant (*red*) conditions. The distribution of data points is displayed as *gray* circles, together with the mean (+) and standard deviation (*whiskers*) for antero-posterior (AP) / medio-lateral (ML) directions and condition. Significant differences at *p* < 0.05 between conditions are marked with asterisk (*)

A significant effect of surface stability was further found for the magnitude of hip movement (Fig. 4). NPL in medio-lateral direction was found to be significantly higher on compliant surface than on firm surface (15.99 ± 18.09 *mG/s*, *p* = 0.001, *d* = 0.88), whereas no differences between conditions were found for antero-posterior direction (20.53 ± 28.32 *mG/s*, *p* = 0.28, *d* = 0.72) and vertical directions (2.83 ± 5.77 *mG/s*, *p* = 0.52, *d* = 0.49).

### Posturography

**Fig. 5:**
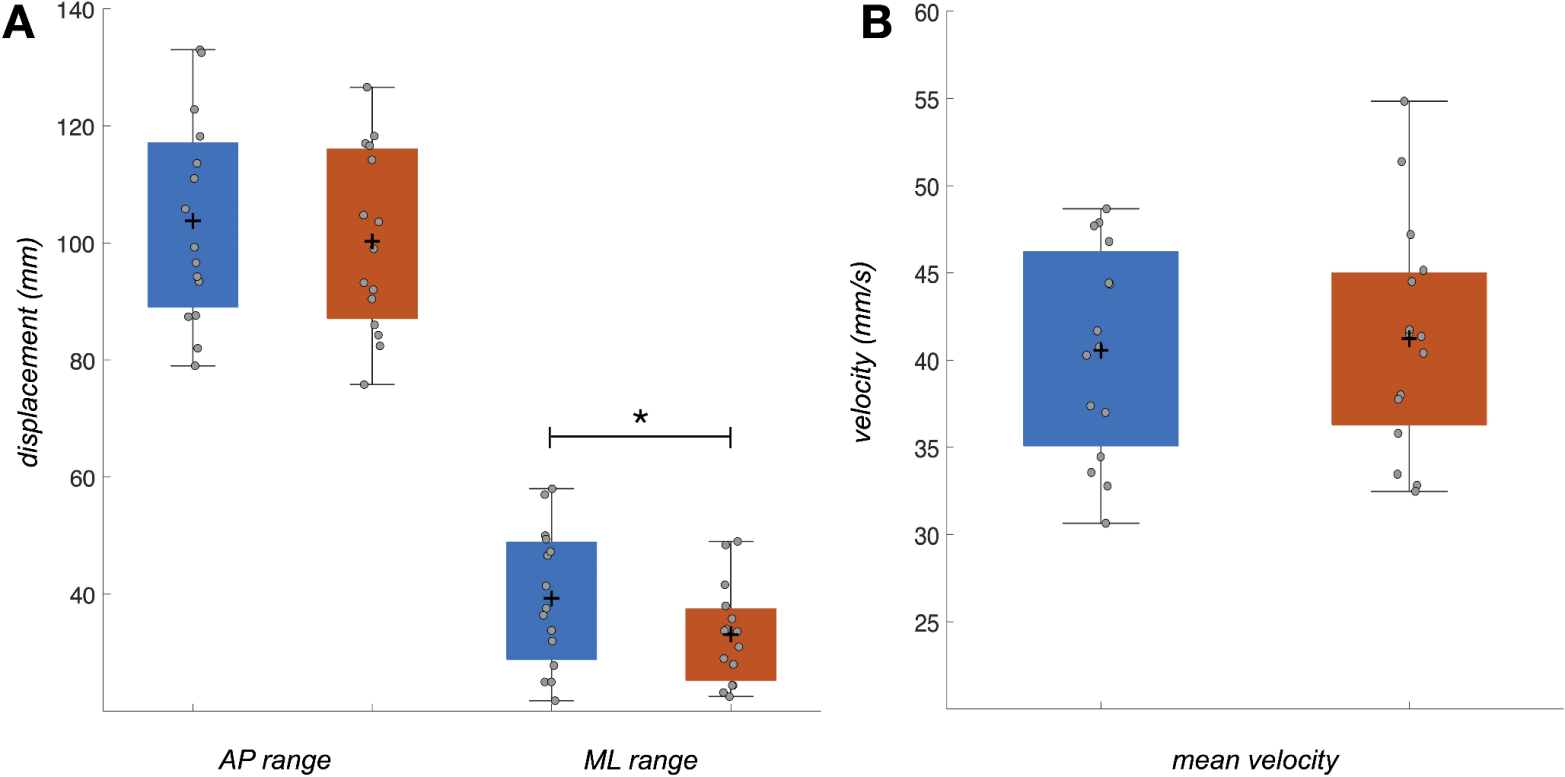
Postural sway, quantified by the center of pressure **A**) antero-posterior (*AP*) and medio-lateral (*ML*) range, as well as **B**) mean velocity under firm (*blue*) and compliant (*red*) conditions. The distribution of individual data samples is shown as *gray* circles, together with the mean (+) and standard deviation (*whiskers*) for each metric and condition. Significant differences at *p* < 0.05 between conditions are marked with asterisk (*)

As shown in Fig. 5, subjects demonstrated significantly lower CoP displacement in the ML range (-6.16 ± 6.41 *mm*, *p* < 0.001, *d* = 0.96) in the compliant compared to the firm condition. No significant differences were found for AP range (-3.49 ± 17.16 *mm*, *p* = 0.40, *d* = 0.20) and mean sway velocity (0.67 ± 4.36 *mm/s*, *p* = 0.53, *d* = 0.15).

### Electromyography

**Fig. 6:**
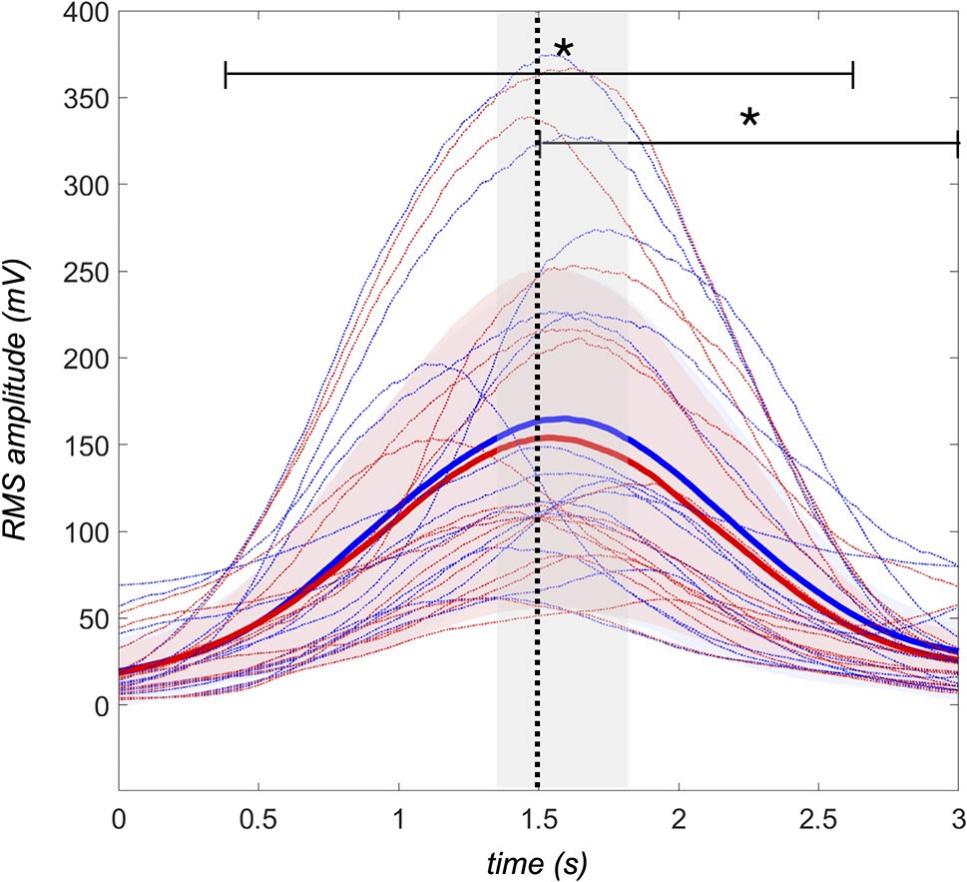
Root mean square (RMS) EMG activity (*mV*) of the rectus femoris under firm (*blue line*) and compliant (*red line*) conditions. The dotted line separates eccentric and concentric phases, with timing variations shown as shade. The mean EMG activity is displayed as solid lines, together with the standard deviation (*shade*) over time. Significant differences at *p* < 0.05 between conditions are marked with asterisk (*)

The mean of both rectus femoris muscles (Fig. 6) showed significantly lower RMS EMG activity on compliant surface (-6.62 ± 11.39 *mV*, *p* = 0.02, *d* = 0.58) over the entire trial length, as well as for the split concentric phase (-9.66 ± 16.07 *mV*, *p* = 0.02, *d* = 0.60). No significant difference between conditions was found for the eccentric phase (-3.58 ± 14.01 *mV*, *p* = 0.29, *d* = 0.25).

### Electroencephalography

**Fig. 7:**
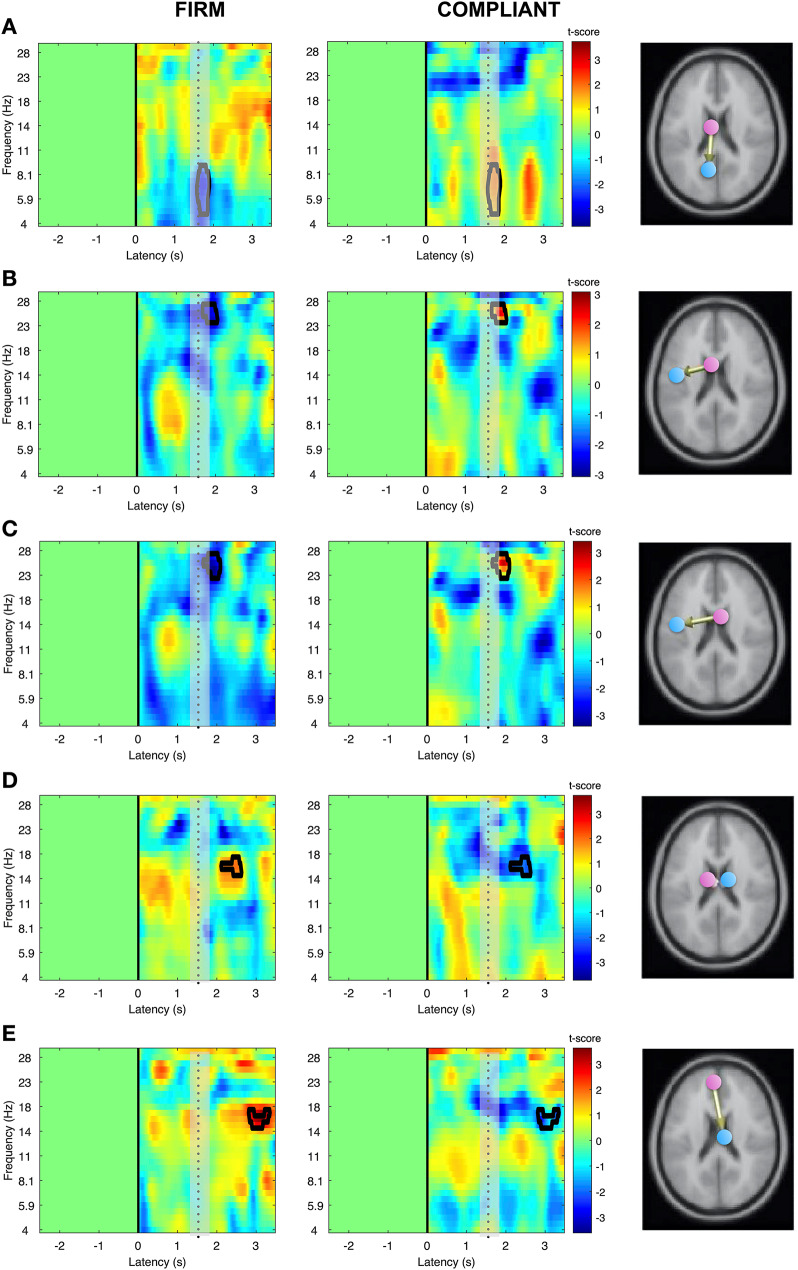
Time-frequency plots of effective connectivity patterns (represented as t-statistics), showing significant differences at *p* < 0.01 (black marking) for the eccentric and concentric phase (separated by dotted line) of the bodyweight squat on firm (left column) and compliant (right column) surface, depicted for estimated significant edges: cingulum mid left to precuneus left (**A**), supplementary motor left to precentral left B), supplementary motor right to precentral left (**C**), cingulum mid left to cingulum mid right (**D**), as well as frontal superior medial left to cingulum mid right (**E**)

**Fig. 8:**
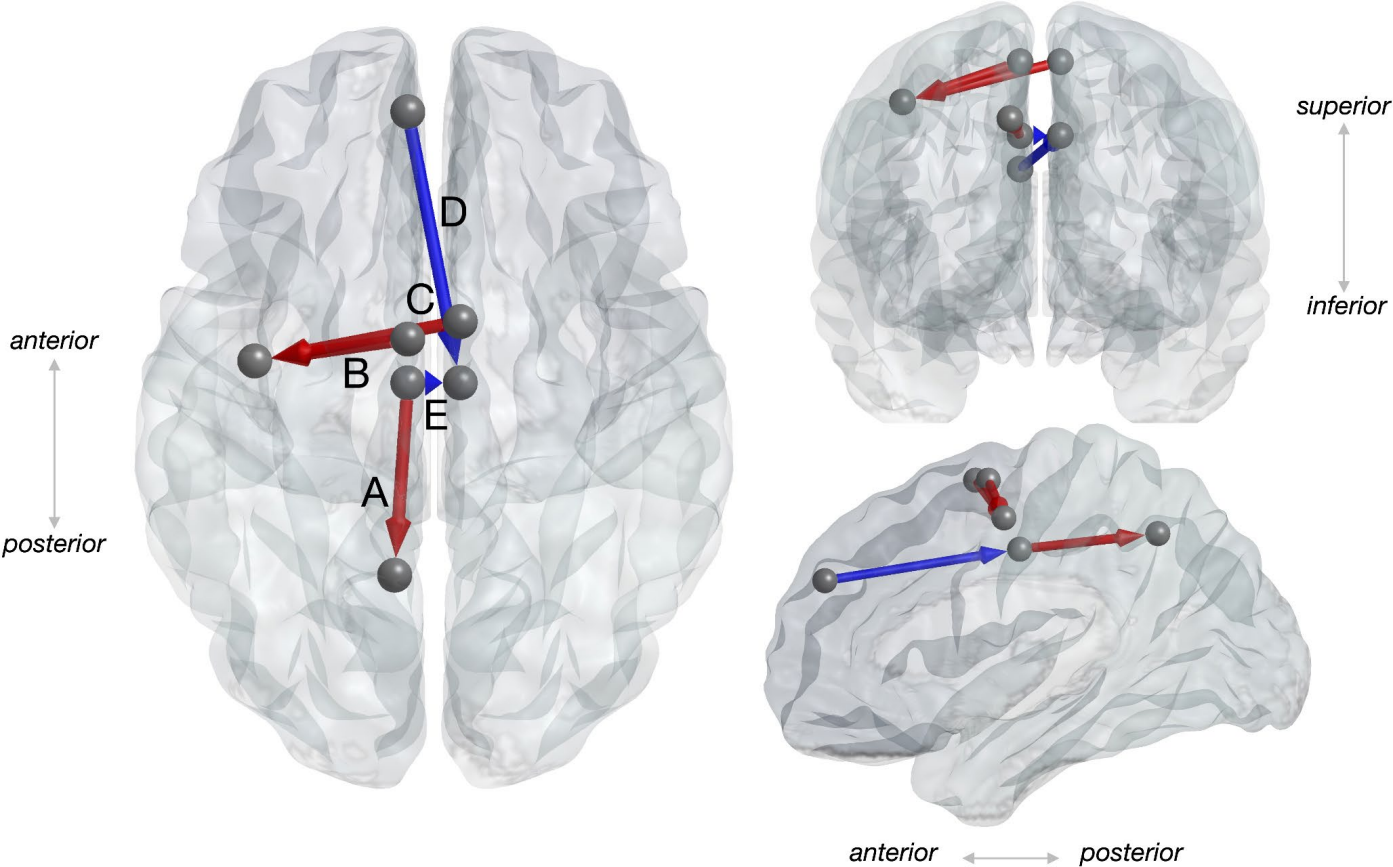
Summary of effective connectivity differences between firm and compliant surface. The compliant surface exhibited greater information flow along edges connecting cingulum mid left to precuneus left (**A**), supplementary motor left to precentral left (**B**) and supplementary motor right to precentral left (**C**), but significantly lower information flow from cingulum mid left to cingulum mid right (**D**) and frontal superior medial left to cingulum mid right (**E**). Arrows represent the direction of information flow, while colors indicate stronger (*red*) or weaker (*blue*) effective connectivity comparing compliant to firm surface. The brain model was generated using BrainNet Viewer software (Xia et al. 2013)

Significantly higher rPDC in the compliant condition (*p* < 0.01) was found for edges connecting cingulum mid left to precuneus left (theta, Fig. 7A), supplementary motor left to precentral left (beta, Fig. 7B), supplementary motor right to precentral left (beta, Fig. 7C). In contrast, significantly lower rPDC in the compliant condition (*p* < 0.01) was found for edges connecting cingulum mid left to cingulum mid right (beta, Fig. 7D), as well as frontal superior medial left to cingulum mid right (beta, Fig. 7E). All significant differences were found for the in the concentric phase (1.5–3 s) of the movement, but not for the eccentric phase (0–1.5 s) Fig. 8.

## Discussion

The aim of the present study was to explore the effects of compliant surface on postural stability and cortical effective connectivity during multi-joint compound movement. While the displacement of neither hip nor CoP in the antero-posterior direction differed between conditions, mean velocity and normalized path length of hip motion significantly increased in the medio-lateral direction on compliant surface. Opposingly, significantly lower CoP displacement in the medio-lateral direction could be observed on the compliant compared to firm surface. Moreover, surface instability led to significant modulations of effective connectivity among sensorimotor nodes, as well as significantly lower rectus femoris activity, predominantly found in the concentric phase of the bodyweight squat.

Multi-joint compound movement on compliant surface place the human body in an unstable configuration, requiring a dynamic integration of multimodal sensory information about the actual positioning and acceleration of body segments (Peterka 2002; Kuo 2005). Whereas earliest postural responses are typically governed by spinal circuits, the dynamic weighting of proprioceptive information, as well as the modulation of corrective motor actions in situations of postural instability is attributed to sensorimotor networks on the cortical level (Schut et al. 2017; Peterka 2018; Alix-Fages et al. 2022). In that regard, the present study utilized mobile neuroimaging to explore the effects of postural instability on cortical effective connectivity during bodyweight squats. As a result, significant modulations of causal information flow could be observed in the concentric movement phase on compliant surface among edges connecting nodes allocated to medial prefrontal, (supplementary) motor and postcentral / parietal areas of the brain. Wittenberg and colleagues (2017) have previously shown that these areas are involved in processes for controlling posture in upright stance and might thus form a postural control network which shows transient reorganization as a response to postural instability (Mierau et al. 2017; Varghese et al. 2019). In the present study, frequency-specific modulations of effective connectivity were observed for short-range connections among neighboring nodes previously associated with postural control. Furthermore, these amendments showed frequency-specific characteristics particularly in the theta and beta bands. Previous studies have associated a theta network among sensorimotor edges with somatosensory information processing for executing postural responses (Mierau et al. 2017; Varghese et al. 2019). Within this theta frequency band, transient connections between adjacent nodes in the fronto-central and posterior parietal cortex, estimated around supplementary motor area and primary motor cortex, were shown to increase connectivity strength in response to perturbations of the supporting surface (Mierau et al. 2017; Varghese et al. 2019; Solis-Escalante et al. 2020). Similarly, the present findings revealed a distinctive increase of effective connectivity within theta frequencies between nodes allocated to medial motor (cingulum) and medial superior parietal (precuneus) areas immediately after initiation of the concentric movement phase on compliant surface. This enhanced information flow related to the time period of vertical body acceleration might therefore represent a mechanism signaling the necessity to increase top-down cognitive control and the evaluation of expected sensory feedback with that generated through the motor action on compliant surface for updating sensory-motor integration processes (Narayana et al. 2013; Cavanagh and Frank 2014; Nakamura et al. 2021; Watanabe et al. 2021). However, theta-band oscillations may not only mediate connectivity in regions involved in cognitive processes, but also modulate processes supporting the interaction between higher-level cognitive processes and motor control systems (Watanabe et al. 2021).

Within the same temporal window as the increased theta band information flow was found (onset concentric phase), a significant increase of beta band effective connectivity was present in motor-related edges (supplementary motor area, left precentral cortex). The beta frequency band is recognized for its strong association with voluntary muscle activation across motor tasks (Khanna and Carmena 2015). Previous research has found connectivity of motor areas within the beta frequency band in close temporal association with reactive postural responses (Varghese et al. 2019). In the interval immediately following the onset of the concentric movement, vertical ground reaction forces consequently unfold their mechanical effect of surface instability. Therefore, the increased information flow within different effector areas (upper / lower extremity) may point to adaptive processes of whole-body action planning to cope with instantaneous postural instability (Wagner et al. 2016; Gordon et al. 2023). Interestingly, the corresponding motor edges only concerned a lateral precentral node within the left hemisphere, while no significant motor edges were found for the right hemisphere. It may thus be speculated that a task-dependent association of the motor cortex in the left hemisphere could be indicative for a functional asymmetry between the two homologous primary motor areas for controlling preparatory postural responses at the onset of the concentric compound movement on compliant surface (Cioncoloni et al. 2016; Noé et al. 2022).

Lastly, beta effective connectivity in motor and fronto-central edges (cingulum, frontal superior medial) was significantly higher in later stages, as well as after termination of the squat movement in the firm surface condition. In general, the beta frequency band has also been associated with modulations of somatosensory responsiveness (Pfurtscheller and Lopes 1999; Kilavik et al. 2013; Nakamura et al. 2021). As such, sensorimotor circuits respond to exogeneous sensory stimulation and mediate inhibitory or excitatory motor pathways through information transmitted in the beta frequency range (Picazio et al. 2014; Wessel 2018). Thus, interactions between functionally specialized frontal and motor areas may determine the current state of sensorimotor networks for actively governing cortico-muscular interactions (Huang et al. 2016; Kenville et al. 2020a; Nakamura et al. 2021). Especially in presence of elevated medio-lateral hip motion in the concentric phase of the bodyweight squat, active cortical contributions appear to be required for dealing with persistent instability (Slobounov et al. 2008; Nandi et al. 2018).

Correspondingly, Feige and colleagues (2000) have found that strong synchronization between cortical areas and activated muscles in the beta frequency band indicated a transition into a new equilibrium state after movement execution. Thus, weaker connectivity in the termination period of the bodyweight squat, in line with increased hip movement, may point to a less efficient transition from an unstable to a stable configuration of the postural system on compliant compared to firm surface (Kristeva et al. 2007). Further elaboration of cortico-muscular interactions may help to better understand the effects of sensory noise on cortico-spinal network dynamics (Kenville et al. 2020a).

Another finding of the current study was the reduced rectus femoris activity on compliant compared to firm surface. While the eccentric phase did not show any differences between conditions, recuts femoris activity significantly decreased from firm to compliant surface in the concentric phase of the movement. Promsri (2022) already demonstrated that muscle activity of the rectus femoris muscle was reduced while balancing on an unstable surface compared to a stable surface. Additionally, lower EMG activity of the prime movers, such as the recuts femoris, has already been observed in squats on inflated discs, BOSU balls or balance cones compared with firm surface (McBride et al. 2010; Saeterbakken and Fimland 2013). As previously shown, reduced involvement of prime movers may point to greater involvement of the vastus lateralis, vastus medialis and biceps femoris in the presence of surface instability (Hyong and Kang 2013; Buscà et al. 2020), serving as an adaptive strategy of the sensorimotor system to support limb stiffness when confronted with elevated stability constraints (Gribble et al. 2003; Faisal et al. 2008; Kenville et al. 2020b). Owing the adaptive nature of postural control, encoded synergies within parallel and hierarchical muscle networks may facilitate to reduce the dimensionality of postural sway for ultimately achieving a stable stance at the time of vertical force generation (Boonstra et al. 2015; Singh et al. 2018; Munoz-Martel et al. 2019).

In the present study, compliant surface particularly affected control of hip motion during bodyweight squats in terms of higher medio-lateral acceleration and difficulties timing the onset of the concentric movement. Previous findings have shown that increased medio-lateral sway of the hip was evident in bipedal stance on foam surface (Alqahtani et al. 2020). Due to the mechanical fluctuations of the supporting surface, reliability of proprioceptive feedback from ankle receptors is reduced in this condition (van den Bogaart et al. 2022). During compound movements, mechanically unstable surface conditions were linked with higher eversion and simultaneously reduced dorsiflexion of the ankle joint (Nairn et al. 2017). While antero-posterior stability is predominantly maintained via these plantar/dorsiflexor mechanisms, the effectiveness of ankle-generated moments for postural control is limited under compliant surface conditions (Winter et al. 1996; van den Bogaart et al. 2022). On that account, compliant surface may inherently necessitate a stricter stiffness of the ankle joint in order to cope with the mechanically unstable surface characteristics of the foam (Kiers et al. 2012). As randomness of mechanical surface instability accumulates noisy somatosensory information from lower limb receptors (Faisal et al. 2008; van Dieën et al. 2015), postural stability based on distal joint actions might be less effective to control the center of mass during movement execution (Otten 1999; Kiers et al. 2012). Therefore, surface compliance may lead to a shift from a distal ankle to a more proximal hip strategy for maintaining postural equilibrium, particularly necessitating weight-shifting between legs and counter-rotations driven by hip abductor/adductor or trunk lateroflexor muscles that result in increased medio-lateral sway (Winter et al. 1996; van Dieën et al. 2015; van den Bogaart et al. 2022). Interestingly, while several studies have reported elevated sway and velocity on different unstable surfaces (Gebel et al. 2020; Büchel et al. 2021), the potential switch of the dominant postural control strategy in the present study only resulted in more rapid medio-lateral hip movement, but concurrently decreased CoP displacement in this direction (Whitney et al. 2011). Speculatively aligning with the current EMG findings, it is conceivable that modified muscle activity in the lower limb contributed to the stabilization of the knee joint at the expense of diminished hip control (Boonstra et al. 2015; Singh et al. 2018; Munoz-Martel et al. 2019). However, further research employing more complex and holistic biomechanical and electromyographic analyses is needed to better understand the effects of compliant surfaces on intricate body dynamics.

Altogether, the compliant surface led to increased difficulties controlling hip motion in the medio-lateral plane and consequently timing the concentric movement. Moreover, a decreased activation of the prime movers accompanied by modulations of effective brain connectivity among fronto-central nodes may point to altered demands on sensorimotor information processing in presence of sensory noise during bodyweight squat movement on compliant surface.

## Methodological considerations

The current study used a multimodal mobile brain and body imaging approach to link cortical causal information flow with concomitant motor behavior during bodyweight squats. However, certain limitations inherent to this methodological procedure are crucial to acknowledge for contextualizing the present findings.

For standardizing movement execution across subjects, timing and speed were visually paced using a digital cue displayed on a ground-level screen. In contrast to this, the natural speed of a squat movement could inherently vary depending on individual factors, and participants may subsequently adapt their motor behavior in relation to external demands (Frost et al. 2015). Hence, motor and neurophysiological observations provided by the given protocol might be influenced by a degree of artificial movement behavior. Although, the consistency of trunk and knee flexion angles across subjects were not controlled, a strong correlation between negative displacement and body height may suggest consistent squat mechanics relative to the anthropometrics of the subjects (Zawadka et al. 2020). Nevertheless, robust analysis of movement-related neural dynamics require equivalent data epochs across trials / subjects for revealing underlying dynamics, which was successfully demonstrated in a recent study by Kenville et al. (2020a).

Another methodological aspect potentially confounding the neurophysiological data could be related to leg dominance of the participants, as the study sample was composed of six left and nine right dominant individuals. Although the bodyweight squats were performed in a bipedal manner, a potential effect of leg or hemispheric dominance on postural stability could not be completely excluded (Promsri et al. 2020, 2023). In turn, motor strategies and muscular synergies might be different between the dominant and non-dominant lower limbs. Therefore, increasing the number of EMG sensors and controlling for limb dominance might help to better understand laterality-dependent patterns observed in the EMG and EEG data.

Ultimately, several factors of the present EEG data processing approach may have influenced the findings. (1) The stringent selection of frequently occurring functional ICs across subjects may have resulted in the exclusion of other cortical sources associated with sensorimotor processes relevant to the bodyweight squat, which could potentially create an incomplete representation of the actual dynamics within the underlying cortical network. Essentially, the reason for choosing this procedure was to resolve the variability of estimated sources across subjects and the assumption that stability of MVAR models depends on similar input parameters per subject (Jurgiel et al. 2021). As postural control likely relies on topographically delimited functional networks within the cortex (Mierau et al. 2017), the IC selection was deemed to build upon previously reported cortical sources of activity involved in postural control processes (Wittenberg et al. 2017; Gebel et al. 2020; Büchel et al. 2021). Additionally, (2) source space analysis based on ICA assumes mutual independence of underlying sources, which theoretically contradicts causality between ICs. However, the independence of decomposed ICs is based on instantaneous dependencies between time series. While rPDC– as a measure of effective connectivity - estimates causality between past information of one and the current information of another node, both approaches are practically not conflicting (Coben and Mohammad-Rezazadeh 2015). Nevertheless, the validity of effective connectivity dynamics is reliant on fitting a stable MVAR model. Thus, the model ideally passes whiteness, consistency and stability requirements in order to avoid overfitting (Lütkepohl 2005). In the current study, (3) the residuals of the computed model closely failed the autocorrelation function (ACF) whiteness test (with *p* = 0.80 < 0.95), suggesting that the model might not entirely capture the underlying data structure. As a consequence, the small departures from white noise properties in residuals may indicate omitted variables, misspecification of the model or other issues that can affect the reliability of model results (Coben and Mohammad-Rezazadeh 2015). In contrast, consistency (> 85%) and stability tests (with *idx* = -0.07 < 0) passed the requirements for appropriate model fit and may though allow the computed MVAR model to account for the vast majority of the observed dynamics (Coben and Mohammad-Rezazadeh 2015; Courellis et al. 2017).

## Conclusion

In conclusion, the present findings revealed novel insights into brain and body dynamics during multi-joint compound movement on compliant surface. The compliant surface seemed to elicit difficulties controlling hip motion in the medio-lateral plane and the timing of concentric movement initiation. Moreover, decreased activation of the prime mover muscles was accompanied by modulations of effective brain connectivity within fronto-central edges of the brain. These findings may therefore point to altered demands on sensorimotor information processing in presence of sensory noise and temporal postural instability resulting from a compliant support surface. Consequently, exercise combined with surface instability may enhance neural information processing and ultimately facilitate previously proposed effects of improved multi-joint coordination, muscular co-activations and anticipatory postural adjustments. However, further studies are needed to elaborate these findings and evaluate a potential benefit for athletic and clinical populations.

## Data Availability

No datasets were generated or analysed during the current study.
